# Achievement of adequate nutrition contributes to maintaining the skeletal muscle area in patients with sepsis undergoing early mobilization: a retrospective observational study

**DOI:** 10.1186/s40795-024-00846-w

**Published:** 2024-02-24

**Authors:** Ryo Abe, Takashi Shimazui, Masayuki Sugo, Akihiro Ogawa, Michito Namekawa, Nobuya Kitamura, Satoshi Kido

**Affiliations:** 1 Department of Rehabilitation, Kimitsu Chuo Hospital, 1010 Sakurai, Kisarazu, Chiba 292-0822 Japan; 2 Department of Health and Social Services, Graduate School of Saitama Prefectural University, Koshigaya, Saitama Japan; 3 Department of Emergency and Critical Care Medicine, Kimitsu Chuo Hospital, Kisarazu, Japan

**Keywords:** Sepsis, Intensive care unit-acquired weakness, Skeletal muscle area, Nutrition, Energy achievement rate

## Abstract

**Background:**

The onset of muscle loss in critically ill patients, known as intensive care unit-acquired weakness (ICU-AW), worsens their outcomes. Preventing muscle loss, which begins in the early phase of critical illness, is crucial in patient care. Adequate nutrition management may contribute to maintaining muscles; however, its evidence in patients with sepsis is insufficient. This study aimed to analyze the association between energy achievement rate in the first 7-days of critical care and muscle area changes evaluated by computed tomography (CT).

**Methods:**

This was a retrospective observational study. Patients with sepsis admitted to the intensive care (ICU) of a tertiary care hospital in Japan were included. They were divided into three groups according to tertiles of the first 7-day energy achievement rate calculated using administered energy doses and basement energy expenditure. Skeletal muscle area (SMA) and changes in SMA were determined by CT on ICU admission and within days 7–10 of ICU admission. SMA maintenance was defined as SMA change ≥ 100%. Logistic regression analyses were performed to analyze the association of energy achievement rate with SMA changes (primary outcome) and in-hospital 28-day mortality (secondary outcome).

**Results:**

Patients (*n* = 93) were classified into low, middle, and high groups according to their 7-day energy achievement rate (median rates, 16.8%, 38.8%, and 73.4%, respectively). The CT scans showed that SMA decreased between the CT scans in the low and middle groups, whereas it was maintained in the high group (median changes, -8.5%, -11.7%, and 2.8%, respectively). Univariate and multivariate logistic regression analyses showed that high energy achievement rate was significantly associated with SMA maintenance (reference, middle energy achieved group; univariate, odds ratio [95% confidence interval] 6.23 [2.04–19.10], *P* = 0.0013; multivariate, odds ratio [95% confidence interval] 5.92 [1.90–18.40], *P* = 0.0021). There was no significant difference in the association between energy achievement rate and mortality among the three groups.

**Conclusions:**

Our study found that a fulfillment of energy achievement in the first 7 days of hospitalization was associated with maintenance of muscle area. Thus, satisfying adequate energy should be considered even in patients with sepsis.

**Supplementary Information:**

The online version contains supplementary material available at 10.1186/s40795-024-00846-w.

## Background

Sepsis, the leading condition requiring intensive care unit (ICU) treatment [1], has high mortality and poor prognosis, including long-term muscle disabling or sarcopenia [2, 3]. Muscle loss caused by acute catabolic reactions and coincident critical illness-induced polyneuropathy/myopathy is described as intensive care unit-acquired weakness (ICU-AW) [4]. Among the factors that contribute to ICU-AW, muscle loss is particularly important and has been associated with increased mortality, prolonged ventilation, and functional decline in survivors [3, 5]. Preventing muscle loss in patients with sepsis might play an important role in improving their prognoses.

Adequate nutritional support is essential in preventing muscle loss in critically ill patients. It has been reported that the energy achievement rate ≥ 60% may contribute to muscle area maintenance in patients admitted to the ICU [6, 7]. Along with maintaining muscle area, the satisfaction of the energy requirement in the early phase of critical illness may contribute to improving long-term physiological conditions and reducing the sarcopenia incidence [8]. However, despite the beneficial roles of satisfying energy requirements in critically ill patients, the energy achievement rate in the early phase of sepsis tends to be lowered [9]. Since a previous study indicated that overnutrition in the early phase of sepsis was associated with poor outcomes, including death [10, 11], and there is a lack of knowledge of the risk association between low energy achievement and muscle area loss, physicians might pay less attention to satisfying the energy requirement in the early phase of sepsis. Clarifying the association and determining the significance of energy achievement even in patients with sepsis may contribute to improving the quality of sepsis care.

In this study, we hypothesized that fulfillment of energy requirements contributes to maintaining muscle area in patients with sepsis. Therefore, we analyzed the associations between energy achievement rate and skeletal muscle area (SMA) in patients with sepsis who underwent early mobilization.

## Methods

### Study design

This was a retrospective observational study conducted in a Japanese tertiary hospital. Adult patients with sepsis (age ≥ 18 years) who were admitted to the ICU of Kimitsu Chuo Hospital from January 2016 to August 2021 were screened. Patients were enrolled if they had been admitted to the ICU for at least 72 h, undergone computed tomography (CT) imaging on ICU admission (1st CT) and within day 7–10 of ICU admission (2nd CT), and initiated early mobilization within 48 h from ICU admission. Patients were excluded if they did not receive any nutritional therapy during their ICU admission, had missing data on their body weight, or had a history of neuromuscular or cerebrovascular disease. We included the sample size deemed sufficient for analysis, according to previous reports that investigated the associations between energy achievement rate and outcomes in critically ill patients (association between muscle area loss, *n* = 23; mortality, *n* = 117) [6, 11].

This study was approved by the ethics committee of Kimitsu Chuo Hospital (approval number 584). Since this observational research used retrospective medical records, we did not obtain written or oral consent from the participants. However, to ensure that the participants or their surrogates had the opportunity to decide whether they wished to refuse to participate in the study or not, the opt-out information was posted on the Kimitsu Chuo Hospital home page. Patient data were immediately discarded if they refused to participate in the study.

### Data collection and definition

Data on baseline characteristics including age, sex, body mass index (BMI), comorbidity (malignancy; hypertension; diabetes mellitus [DM]; and chronic organ dysfunctions [heart failure, chronic kidney disease, chronic liver disease, and chronic lung disease]), charlson comorbidity index (CCI), severity score (acute physiologic and chronic health evaluation II [APACHE II score], sequential organ failure assessment [SOFA score] on admission), modified nutritional risk assessment score (mNUTRIC score), presence of septic shock, steroid use before ICU admission, etiology of sepsis (pneumonia/urinary tract infection/intra-abdominal infection/other), SMA evaluated using 1st CT and 2nd CT, and nutritional intervention and categorization (parenteral nutrition [PN; total parenteral nutrition {TPN} and peripheral parenteral nutrition {PPN}], enteral nutrition [EN; gastric tube feeding, or jejunal tube feeding]) were retrieved. Data on changes in SMA during the 1st and 2nd CT scans and in-hospital 28-day mortality were retrieved as outcome values.

CT was selected to evaluate muscle area because of our study design, and it is considered the standard method for assessing skeletal muscle in ICU patients as it accurately reflects the SMA of the whole body (Pearson’s correlation, *r* = 0.9) [12, 13]. CT on the body trunk is generally examined to search for infectious sources in clinical practice [14]; thus, it was suitable for retrieving data retrospectively. SMA was calculated as the total area of rectus abdominis/transverse abdominal/oblique abdominis/psoas/paraspinal muscles [15]. These muscle areas were evaluated at the CT slice level, where the entire third lumbar transverse process was visible, and the Hounsfield Unit among the regions of interest was − 29 to + 150 [15]. The evaluation was performed by a Japan Radiological Society-certified radiological technologist. The percentage of changes in SMA was calculated as follows: ([SMA at the 2nd CT / SMA at the 1st CT] *100). A 3D medical imaging workstation (Ziostation2) software was used for image analysis. The primary outcome involved assessing the maintenance of muscle area on an inter-individual basis without adjusting for factors such as body weight [16].

Daily energy achievement rate was calculated as follows: total energy dose administered to each patient/basement energy expenditure (BEE) calculated using the Harris-Benedict formula (Total Energy / Total BEE) [17]. The achievement rate for energy intake over 7 days, as well as the supply levels of energy and protein, were also calculated. Due to the lack of an established cut-off value of adequate energy achievement rate, we classified the patients into three groups according to tertiles of the 7-day energy achievement rate (low, middle, and high groups) to avoid the selection bias and evaluated the association between energy achievement rate and outcomes. The nutrition therapy was not protocolized; initiating enteral and/or parental nutrition was chosen at the physician’s discretion, and the amount of nutrition was increased. SMA change ≥ 100% indicated SMA maintenance. The intervals between the 1st and 2nd CT scans (CT interval) were calculated to evaluate differences between the groups. Sepsis and septic shock were diagnosed based on the Sepsis-3 criteria [1].

### Statistical analysis

The primary outcome was SMA change during the CT scans. The secondary outcome was in-hospital 28-day mortality. The primary analysis was logistic regression analysis for the association between energy achievement rate and outcomes. Age, sex, and SOFA score on admission were included as variables in the multivariate analysis. The middle energy achieved group was used as a reference to analyze the contribution of low or high energy achievement to the outcomes. To further adjust the impact of illness severity and the significance of macronutrition on the outcome, sensitivity analysis with additional covariates, including septic shock and 7-day protein supply, were analyzed. Moreover, sensitivity analysis between the groups with daily energy achievement rate ≥ 70% and < 70% was performed according to the guideline recommendation of energy achievement rate [18]. Furthermore, since geriatric patients are prone to loss of muscles and likely to have sarcopenia as a result of muscle loss, which is especially associated with the outcomes [19], subgroup analysis in patients aged ≥ 65 years was also performed.

Categorical variables were analyzed using Fisher’s exact test, and continuous variables with the Kruskal–Wallis test. The percentage of SMA change in each group was evaluated using the paired t-test. Data are expressed as median (interquartile range). Two-tailed *P* values < 0.05 were considered significant. The validity of the SMA calculation was evaluated by intra-rater reliability (ICC [1.1]) in 30 randomly selected cases. Analyses were performed using the statistical analysis software R version 4.0.2 (R Foundation for Statistical Computing, Vienna, Austria).

## Results

The intra-rater reliability (ICC [1.1]) for the measurement of SMA was 0.93, indicating that the measurements were reliable.

**Fig. 1:**
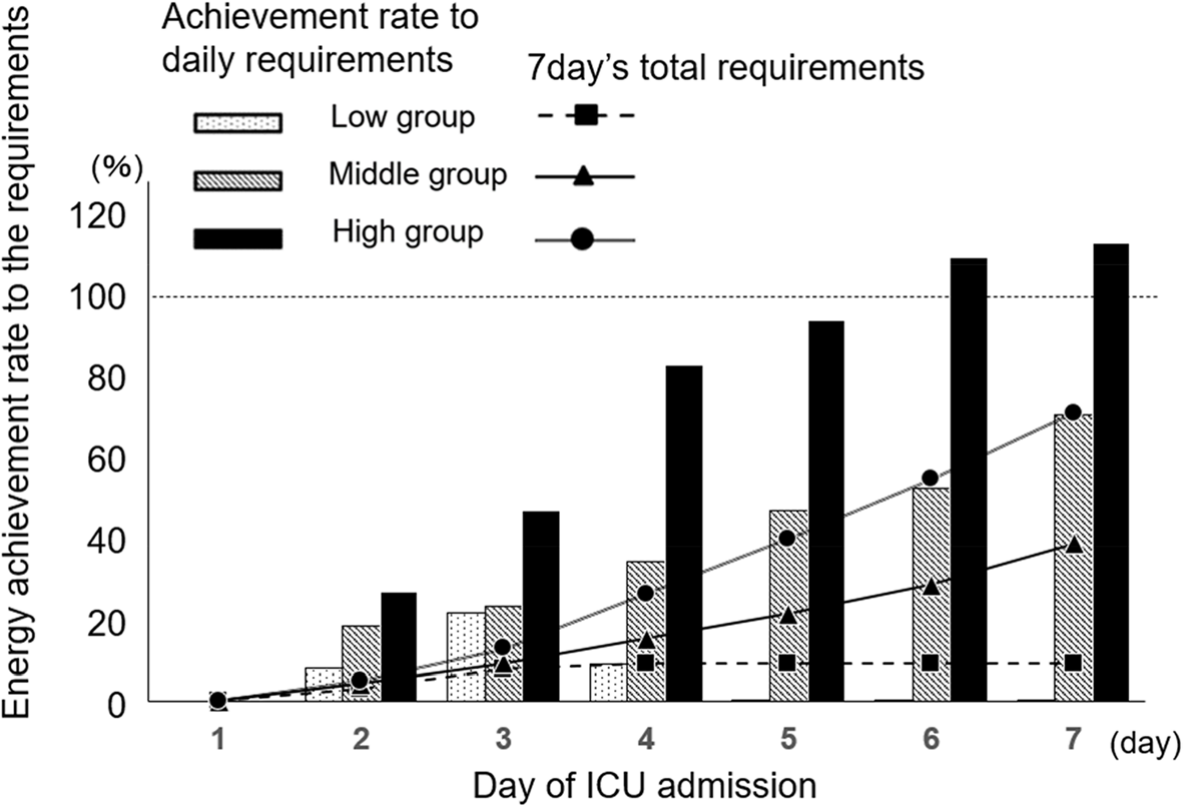
Energy achievement rates for the 7 days of ICU admission. Data are presented as median. ICU, intensive care unit

During the study period, we screened 228 patients with sepsis, 93 of whom were included in the analysis (Supplementary Fig. 1). The median energy achievement rates on the 7-days of ICU admission were 16.8% for the low group (total energy supply, 1500 kcal [range, 810–2025], total protein supply, 67.5 g [range, 48–122]), 38.8% for the middle group (total energy supply, 3156 kcal [range, 2895–3873], total protein supply, 130 g [range, 100–158]), and 73.4% for the high group (total energy supply, 5500 kcal [range, 4334–7277], total protein supply, 209 g [range, 150–279]). While there were no statistical differences in any of the macronutrient supplies, a trend for significance in the protein supply was observed between the groups (carbohydrates, *P* = 0.43; protein, *P* = 0.069; fat, *P* = 0.97; Table 1). The median energy achievement rates on day 7 were 0.0% for the low group, 71.6% for the middle group, and 116.2% for the high group. The energy achievement rate increased over time and exceeded 100% on day 6 in the middle group and on day 7 in the high group, whereas it never exceeded 100% on any day in the low group (Fig. 1). The protein intake had a similar trend over the 7 days (Supplementary Fig. 2). There were no differences in nutritional administration routes between the groups (Table 1).

**Table 1:** Patients’ baseline characteristics and clinical outcomes according to tertiles of the energy achievement rate

	Energy achievement rate			
	Low (*n* = 31)	Middle (*n* = 31)	High (*n* = 31)	*P* value
Characteristic				
7-day energy achievement rate^a^	16.8 (7.8–22.7)	38.8 (35.3–44.0)	73.4 (63.5–87.6)	< 0.0001
7-day energy supply (kcal)	1500 (810–2025)	3156 (2895–3873)	5500 (4334–7277)	< 0.0001
7-day protein supply (g)	67.5 (48–122)	130 (100–158)	209 (150–279)	< 0.0001
Percentage of macronutrients (%)				
Carbohydrates	56 (33–64)	63 (56–63)	63 (34–65)	0.43
Protein	20 (15–34)	19 (15–20)	20 (17–20)	0.069
Fat	24 (15–33)	18 (15–25)	17 (15–25)	0.97
Age, years	77 (71–82)	74 (66–80)	76 (69–83)	0.42
Male sex, n (%)	22 (70.9)	18 (58.1)	15 (48.4)	0.22
BMI on ICU admission (kg/m²)	21.4 (18.4–23.4)	20.5 (19.5–23.7)	20.4 (18.4–21.7)	0.58
SMA on ICU admission (cm²)	107.5 (87.8-123.8)	94.7 (81.2-132.4)	84.9 (68.7–98.3)	0.015
Comorbidity, n (%)				
Malignancy	7 (22.6)	8 (25.8)	3 (9.7)	0.25
Hypertension	11 (35.5)	9 (29.0)	5 (16.1)	0.27
Diabetes mellitus	8 (25.8)	12 (38.7)	10 (32.3)	0.59
Heart failure	4 (12.9)	6 (19.4)	7 (22.6)	0.71
Chronic kidney disease	6 (19.4)	3 (9.7)	2 (6.5)	0.37
Chronic liver disease	1 (3.2)	2 (6.5)	3 (9.7)	0.87
Chronic lung disease	1 (3.2)	3 (9.7)	2 (6.5)	0.87
Charlson comorbidity index	7 (5–7)	6 (4–8)	6 (5–7)	0.57
SOFA score on admission	9 (5–12)	10 (9–13)	9 (6–13)	0.18
APACHE II score	25 (18–28)	26 (22–30)	23 (20–28)	0.85
mNUTRIC score	6 (4–7)	6 (5–7)	5 (5–7)	0.36
Septic shock, n (%)	18 (58.1)	10 (32.3)	13 (41.9)	0.34
Steroid use before admission, n (%)	6 (19.4)	3 (9.7)	4 (12.9)	0.66
Etiology of sepsis, n (%)				
Pneumonia	3 (9.7)	10 (32.3)	6 (19.4)	0.10
Urinary tract infection	17 (54.8)	10 (32.3)	12 (38.7)	0.22
Intra-abdominal infection	5 (16.1)	3 (9.7)	5 (16.1)	0.81
Other	6 (19.4)	8 (25.8)	8 (25.8)	0.87
CT interval (days)	8 (7–10)	8 (7–10)	8 (7–9)	0.75
Nutritional intervention, n (%)				
Parenteral nutrition				
Total parenteral nutrition	9 (29.0)	12 (38.7)	14 (45.2)	0.63
Peripheral parenteral nutrition	4 (12.9)	1 (3.2)	0 (0.0)	0.12
Enteral nutrition				
Gastric tube feeding	14 (45.2)	17 (54.8)	17 (54.8)	0.78
Jejunal tube feeding	4 (12.9)	1 (3.2)	0 (0.0)	0.12
Enteral and parenteral nutrition	11 (35.5)	11 (35.5)	8 (25.8)	0.64
Outcome				
Percent changes in SMA (%)	-8.5 (-15.5 to -1.7)	-11.7 (-18.0 to -1.9)	2.8 (-7.1 to 10.1)	0.0046
In-hospital 28-day mortality, n (%)	7 (22.6)	9 (29.0)	6 (19.4)	0.75

SMA change was evaluated from 1st and 2nd CTs. Values are reported as n; median (interquartile range), or n (%). *P* values were calculated using the Kruskal-Wallis test or Fisher’s exact test

*BMI* Body mass index, *SOFA* score Sequential organ failure assessment score, *APACHE II* Acute physiologic and chronic health evaluation II, *mNUTRIC* Modified nutrition risk in the critically ill, *SMA* Skeletal muscle area, *CT* Computed tomography

^a^Calculated by the formula of total energy dose administered / Harris–Benedict formula from the time of admission to 7 days of illness for each patient

There were no significant differences in comorbidities and CCI, severity scores (SOFA and APACHE II scores), mNUTRIC score, presence of septic shock, steroid use before ICU admission, etiology of sepsis, nutritional intervention, CT interval, and in-hospital 28-day mortality between the groups. Percentage change in SMA was significantly different among the three groups; the low and middle groups showed decreases in SMA, whereas the value was maintained in the high group (changes in SMA, low vs. middle vs. high group, -8.5% vs. -11.7% vs. 2.8%, *P* = 0.0046; Table 1) (Supplementary Fig. 3).

The univariate logistic regression analysis showed that the odds of the high-energy achievement group significantly increased the likelihood of SMA maintenance 6.23-fold more than the middle group (95% CI 2.04–19.10, *P* = 0.0013). However, there was no significant difference for the SMA maintenance in the low energy achievement compared to the middle energy achievement (*P* = 0.39) (Table 2A). Similar to the univariate analysis, multivariate logistic regression analysis adjusted for age, sex, and SOFA score showed that the odds of the high energy achievement group significantly increased the likelihood of SMA maintenance 5.92-fold more than the middle group (95% CI 1.90–18.40, *P* = 0.0021). No statistical difference was found for the SMA maintenance in the low-energy achievement group compared to the middle-energy achievement group (*P* = 0.51) (Table 2B).

**Table 2:** Logistic regression analysis to identify the association between SMA maintenance and energy achievement rate

	Odds ratio	95% Confidence interval	*P* value
A. Univariate analysis			
Energy achievement rate			
Low	1.63	0.53-5.05	0.39
Middle	reference		
High	6.23	2.04-19.10	0.0013
B. Multivariate analysis			
Age	1.02	0.97-1.06	0.51
Male sex	1.66	0.64-4.30	0.29
SOFA score on admission	1.00	0.89-1.13	0.98
Energy achievement rate			
Low	1.50	0.45-5.05	0.51
Middle	reference		
High	5.92	1.90-18.40	0.0021

*SOFA score* Sequential organ failure assessment score, *SMA* Skeletal muscle area

The sensitivity analysis with additional covariables of septic shock and protein supply led to the same conclusion (Supplementary Table 1). Similarly, patients who had an energy achievement rate ≥ 70% significantly maintained SMA, compared with patients who had an energy achievement rate < 70% (Supplementary Table 2A, B). Furthermore, the same results were obtained when only elderly patients aged ≥ 65 years were included (Supplementary Table 3A, B).

There were no significant differences in the risk of in-hospital 28-day mortality between the three groups in both univariate and multivariate analyses (univariate; low, group *P* = 0.56 and high group, *P* = 0.38 and multivariate; low group, *P* = 0.63 and high group, *P* = 0.56; Table 3A, B).

**Table 3:** Logistic regression analysis to identify the association between in-hospital 28-day mortality and energy achievement rate

	Odds ratio	95% Confidence interval	*P* value
A. Univariate analysis			
Energy achievement rate			
Low	1.40	0.47-4.41	0.56
Middle	reference		
High	1.70	0.52-5.55	0.38
B. Multivariate analysis			
Age	0.99	0.95-1.05	0.92
Male sex	5.64	1.46-21.70	0.012
SOFA score on admission	0.87	0.76-0.99	0.047
Energy achievement rate			
Low	1.37	0.38-4.91	0.63
Middle	reference		
High	1.48	0.39-5.50	0.56

*SOFA score* Sequential organ failure assessment score

## Discussion

In the present study, we found that high energy achievement was significantly associated with muscle maintenance but not in-hospital mortality in patients with sepsis.

Energy dosage has been reported to be associated with the maintenance of muscle areas in critically ill patients. In a previous report on critically ill patients admitted to the ICU, energy supplementation with supplemental parenteral nutrition from the 4th day of ICU admission with the goal of 100% energy achievement prevented muscle area loss during the following 5-days, compared with enteral nutrition alone (energy achievement rate, intervention group vs. control group: 99.99% vs. 75.89%, *P* = 0.01) [6]. Additionally, another study found that in the ICU setting, patients receiving high-energy and high-protein supplements showed greater maintenance of quadriceps muscle thickness by day 15 than those receiving standard nutrition (energy achievement rate, intervention group vs. control group: 84% vs. 73%, *P* = 0.01) [20]. In our current study on patients with sepsis, we observed that maintaining muscle area from admission to day 7 is feasible with an energy achievement rate of approximately ≥ 70% (energy achievement rate, low vs. middle vs. high group: 16.8% vs. 38.8% vs. 73.4%, *P* < 0.0001). These findings are consistent with the advised energy achievement rates for critically ill patients according to current guidelines. The decrease in muscle area is linked to an escalation in physical dysfunctions like sarcopenia and higher mortality rates, highlighting the importance of early and adequate nutritional intervention.

There is still controversy about the adequate energy achievement rate in critically ill patients. Guidelines recommend trophic feeding (up to 500 kcal/day) for patients with sepsis in the early stage of the disease [21]. In-hospital mortality was significantly lower in ICU patients whose daily energy achievement rate was targeted at 60–70% of the required intake compared with those whose daily energy achievement rate was targeted at 90–100% [22]. Recent studies also highlight the importance of timing in meeting energy goals. Data indicates that patients reaching their energy targets within the first 3 days of ICU admission had higher 28-day mortality rates compared to those who gradually increased their intake over 10 days [23]. Conversely, a guideline recommends the administration of ≥ 70% energy from day 4 onward since inadequate nutritional intake could cause malnutrition [18]. According to previous reports, overnutrition in the early phase of critical illness may be associated with poor outcomes [24, 25]; however, prolonged underfeeding may be associated with malnutrition. The energy achievement rate in our patients was less than 70% until the third day of ICU admission, even in the high group (Fig. 1), and there was no negative effect on mortality in this group. Although the association between mortality and nutritional treatment strategy should be carefully considered, a satisfying energy achievement rate may not badly affect mortality in patients with sepsis.

Maintenance of SMA is suggested to be associated with decreased mortality and improved physical function after recovery. A previous study on patients requiring ventilatory management reported that 60-day mortality was significantly higher in the group with a 7-day decline in rectus femoris muscle (change from baseline: -14.18%), compared with the group that maintained the muscle (change from baseline: -5.75%) [26]. A study of critically ill patients reported that the physical function associated with muscle strength (grip strength / manual muscle testing results) at ICU discharge was significantly associated with biceps muscle mass on day 7 of ICU admission [27]. Few studies on sepsis have examined the associations between SMA change and outcomes; however, nutritionally adequate treatment should also be considered in the case of sepsis, according to previous reports.

### Strengths and limitations

Our study’s primary novelty lies in the demonstration of the association between energy achievement and muscle area preservation during the early stages of sepsis. The key strength of this research is its specific focus on patients with sepsis, providing unique insights applicable to this demographic.

This study has limitations. First, this is a single-center retrospective study; therefore, several confounding factors may not have been excluded, and treatment and selection bias may have occurred. However, there were no differences in baseline severity score and mNUTRIC score in our cohort, indicating that the patients had similar conditions at baseline. Second, while we estimated the sample size and included enough patients, the sample size was small. Although our results indicated that a high energy achievement rate was not associated with increased mortality, determining non-inferiority requires a greater number of patients. Together with the wide confidence intervals observed in our results, the power of the study, and the source population, the generalization of study results may be limited. Third, the method of nutritional administration was not protocolized. It may have caused variation in the nutrition therapy. Recent studies have suggested the importance of protein supply or micronutrients in the acute phase of critical illness [28, 29]. In addition, patients with more severe condition may not be fed via the EN route or receive insufficient energy until their hemodynamic and blood pressure conditions improve, given that patients with septic shock are more likely to be in the low energy achievement rate group (Table 1). While there were no differences in nutritional administration routes between the groups, indicating that patients received similar treatments even though the energy achievement rates were different between the groups, and the sensitivity analysis adjusted with protein supply and septic shock led to the same conclusion, we could not assess the effect of micronutrients on the outcomes. Fourth, we evaluated muscle area only by CT on the entire third lumbar transverse process. Several other methods, such as ultrasound or CT on the quadriceps muscles, have been reported for the evaluation of the skeletal muscles [13]. Ultrasound can evaluate muscles without suffering ionizing radiation and has high spatial resolution; since sarcopenia is believed to begin from the quadriceps muscle [30, 31], CT evaluation on this muscle can detect early muscle loss, which may indicate long-term physiological deterioration. Fifth, we could not assess the details of the impacts of the treatments on the SMA maintenance. For example, while there was no difference in steroid use between the groups, steroid is known to reduce muscle area in patients with sepsis [32]. Future prospective studies utilizing other methods and considering other covariates will also be considered to evaluate the SMA, aiming to elucidate the relationship between the rate of energy achievement and SMA.

## Conclusion

Satisfying energy requirements during the first 7 days of treatment was associated with SMA maintenance in patients with sepsis who underwent early mobilization. Although the association between mortality should be carefully considered, nutritional sufficiency may support the maintenance of muscle area, and appropriate nutritional management is important even in patients with sepsis.

### Supplementary Information

**Supplementary Material 1.**

**Supplementary Material 2.**

**Supplementary Material 3.**

**Supplementary Material 4.**

**Supplementary Material 5.**

**Supplementary Material 6.**

## Data Availability

The datasets generated and/or analyzed during the current study are available from the corresponding author on reasonable request.

## Abbreviations

APACHE II: Acute physiologic and chronic health evaluation II
BEE: Basement energy expenditure
BMI: Body mass index
CT: Computed tomography
ICU-AW: Intensive care unit acquired weakness
mNUTRIC: Modified nutrition risk in the critically ill
SMA: Skeletal muscle area
SOFA: Sequential organ failure assessment
