# Supplementary material for: Achievement of adequate nutrition contributes to maintaining the skeletal muscle area in patients with sepsis undergoing early mobilization: a retrospective observational study
Source: BMC Nutr. 2024 Feb 24;10:32. doi: 10.1186/s40795-024-00846-w (PMC10893714; doi:10.1186/s40795-024-00846-w)
Supplement: Supplementary file 1 — Supplementary Material 1. [file 40795_2024_846_MOESM1_ESM.pdf]

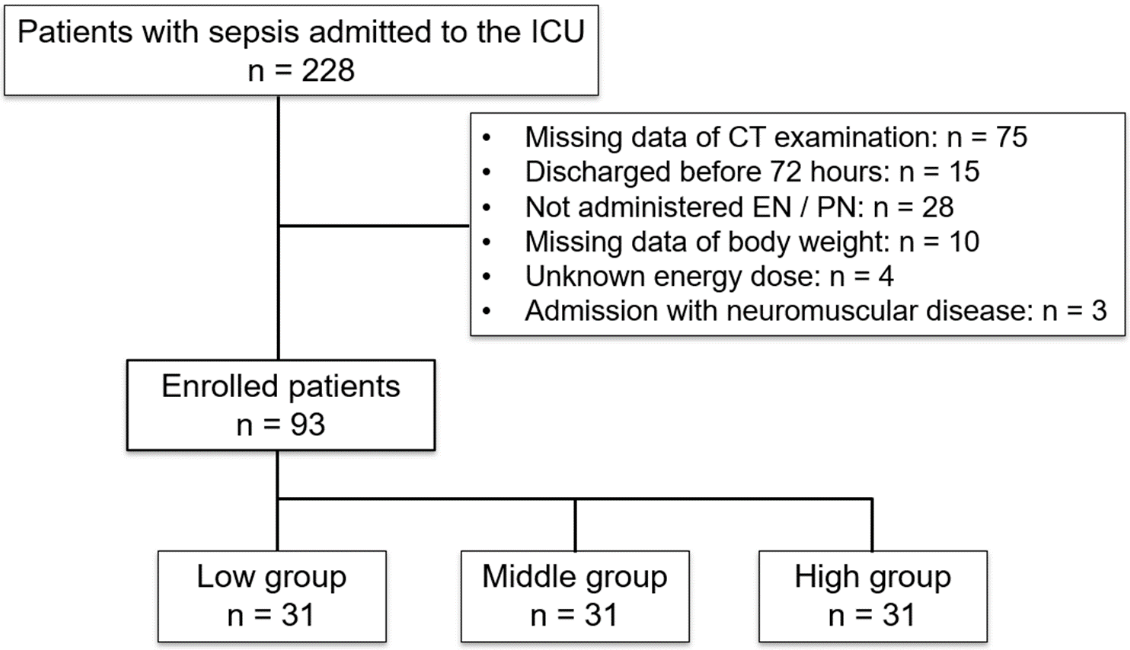

Supplementary Figure 1. Patient flowchart

ICU, intensive care unit; CT, computed tomography; EN, enteral nutrition; PN, parenteral nutrition
