# Supplementary material for: Achievement of adequate nutrition contributes to maintaining the skeletal muscle area in patients with sepsis undergoing early mobilization: a retrospective observational study
Source: BMC Nutr. 2024 Feb 24;10:32. doi: 10.1186/s40795-024-00846-w (PMC10893714; doi:10.1186/s40795-024-00846-w)
Supplement: Supplementary file 3 — Supplementary Material 3. [file 40795_2024_846_MOESM3_ESM.pdf]

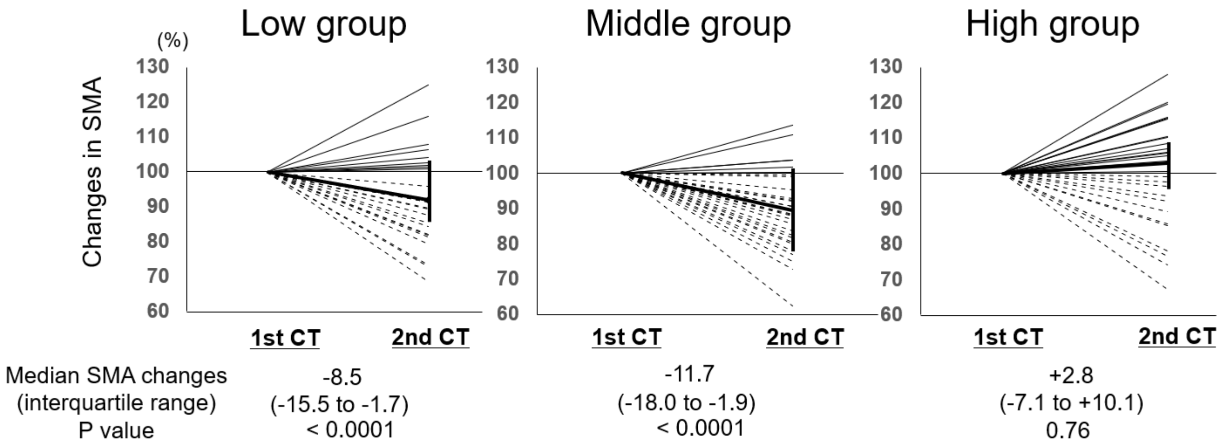

Supplementary Figure 3. Percent changes of SMA from 1st CT to 2nd CT

Bold line indicates median (interquartile range) SMA change of each group. SMA, skeletal muscle area
