# Supplementary material for: Achievement of adequate nutrition contributes to maintaining the skeletal muscle area in patients with sepsis undergoing early mobilization: a retrospective observational study
Source: BMC Nutr. 2024 Feb 24;10:32. doi: 10.1186/s40795-024-00846-w (PMC10893714; doi:10.1186/s40795-024-00846-w)
Supplement: Supplementary file 4 — Supplementary Material 4. [file 40795_2024_846_MOESM4_ESM.pdf]

Supplementary Table 1. Logistic regression analysis to identify the association between SMA maintenance and energy achievement rate, incorporating variables with intergroup differences as covariates

Multivariate analysis

	Odds ratio	95% Confidence interval	P value
Age	1.02	0.97-1.06	0.51
Male sex	1.62	0.62-4.26	0.33
Septic shock	2.57	0.97-6.85	0.059
SOFA score on admission	1.00	0.89-1.13	0.99
7-day Protein supply	1.02	0.96-1.08	0.52
Energy achievement rate			
Low	1.47	0.47-4.63	0.51
Middle	reference		
High	4.99	1.60-15.60	0.0057

SOFA score, sequential organ failure assessment score; SMA, skeletal muscle area
