# Supplementary material for: Achievement of adequate nutrition contributes to maintaining the skeletal muscle area in patients with sepsis undergoing early mobilization: a retrospective observational study
Source: BMC Nutr. 2024 Feb 24;10:32. doi: 10.1186/s40795-024-00846-w (PMC10893714; doi:10.1186/s40795-024-00846-w)
Supplement: Supplementary file 5 — Supplementary Material 5. [file 40795_2024_846_MOESM5_ESM.pdf]

Supplementary Table 2. Logistic regression analysis to identify the association between SMA maintenance energy achievement rates above or below 70%

A. Univariate analysis

	Odds ratio	95% Confidence interval	P value
Energy achievement rate			
70% under	reference		
70% over	7.91	2.35-26.70	< 0.0001

B. Multivariate analysis

	Odds ratio	95% Confidence interval	P value
Age	1.01	0.97-1.06	0.56
Male sex	1.19	0.45-3.16	0.73
SOFA score on admission	1.00	0.89-1.13	0.95
Energy achievement rate			
70% under	reference		
70% over	7.59	2.31-27.00	0.0017

SOFA score, sequential organ failure assessment score; SMA, skeletal muscle area
