# Supplementary material for: Achievement of adequate nutrition contributes to maintaining the skeletal muscle area in patients with sepsis undergoing early mobilization: a retrospective observational study
Source: BMC Nutr. 2024 Feb 24;10:32. doi: 10.1186/s40795-024-00846-w (PMC10893714; doi:10.1186/s40795-024-00846-w)
Supplement: Supplementary file 6 — Supplementary Material 6. [file 40795_2024_846_MOESM6_ESM.pdf]

Supplementary Table 3. Logistic regression analysis to identify the association between SMA maintenance and energy achievement rate for patients ≥ 65 years of age

A. Univariate analysis

	Odds ratio	95% Confidence interval	P value
Energy achievement rate			
Low	1.13	0.33-3.92	0.84
Middle	reference		
High	5.35	1.56-18.40	0.0076

B. Multivariate analysis

	Odds ratio	95% Confidence interval	P value
Age	1.06	0.98-1.14	0.15
Male sex	1.59	0.55-4.56	0.39
SOFA score on admission	1.02	0.89-1.16	0.75
Energy achievement rate			
Low	0.99	0.26-3.78	0.99
Middle	reference		
High	5.42	1.52-19.30	0.0092

SOFA score, sequential organ failure assessment score; SMA, skeletal muscle area
